# The ACCOMPLISH study. A cluster randomised trial on the cost-effectiveness of a multicomponent intervention to improve hand hygiene compliance and reduce healthcare associated infections

**DOI:** 10.1186/1471-2458-11-721

**Published:** 2011-09-24

**Authors:** Vicki Erasmus, Anita Huis, Anke Oenema, Pepijn van Empelen, Matthijs C Boog, Elise HE van Beeck, Suzanne Polinder, Ewout W Steyerberg, Jan Hendrik Richardus, Margreet C Vos, Ed F van Beeck

**Affiliations:** 1 Erasmus Medical Centre, Department of Public Health, Rotterdam, the Netherlands; 2 Radboud University Nijmegen Medical Centre, IQ healthcare, Nijmegen, the Netherlands; 3 Research Group Life Style, TNO, Leiden, the Netherlands; 4 Maastricht University, Department of Health Promotion, Maastricht, the Netherlands; 5 Erasmus Medical Centre, Department of Medical Microbiology & Infectious Diseases, Rotterdam, the Netherlands

## Abstract

**Background:**

Public health authorities have recognized lack of hand hygiene in hospitals as one of the important causes of preventable mortality and morbidity at population level. The implementation strategy *ACCOMPLISH *(Actively Creating COMPLIance Saving Health) targets both individual and environmental determinants of hand hygiene. This study aims to evaluate the cost-effectiveness of a multicomponent implementation strategy aimed at the reduction of healthcare associated infections in Dutch hospital care, by promotion of hand hygiene.

**Methods/design:**

The *ACCOMPLISH *package will be evaluated in a two-arm cluster randomised trial in 16 hospitals in the Netherlands, in one intensive care unit and one surgical ward per hospital.

**Intervention**

A multicomponent package, including e-learning, team training, introduction of electronic alcohol based hand rub dispensers and performance feedback.

**Variables**

The primary outcome measure will be the observed hand hygiene compliance rate, measured at baseline and after 6, 12 and 18 months; as a secondary outcome measure the prevalence of healthcare associated infections will be measured at the same time points. Process indicators of the intervention will be collected pre and post intervention. An ex-post economic evaluation of the *ACCOMPLISH *package from a healthcare perspective will be performed.

**Statistical analysis**

Multilevel analysis, using mixed linear modelling techniques will be conducted to assess the effect of the intervention strategy on the overall compliance rate among healthcare workers and on prevalence of healthcare associated infections. Questionnaires on process indicators will be analysed with multivariable linear regression, and will include both behavioural determinants and determinants of innovation. Cost-effectiveness will be assessed by calculating the incremental cost-effectiveness ratio, defined here as the costs for the intervention divided by the difference in prevalence of healthcare associated infections between the intervention and control group.

**Discussion:**

This study is the first RCT to investigate the effects of a hand hygiene intervention programme on the number of healthcare associated infections, and the first to investigate the cost-effectiveness of such an intervention. In addition, if the ACCOMPLISH package proves successful in improving hand hygiene compliance and lowering the prevalence of healthcare associated infections, the package could be disseminated at (inter)national level.

**Trial registration:**

NTR2448

## Background

Public health authorities have recognized lack of hand hygiene (HH) as one of the important causes of preventable mortality and morbidity at population level [1-3]. Both at national (i.e. UK, USA, The Netherlands) and international levels (World Health Organisation) poor HH has been identified as a threat to public health. HH compliance rates are universally low, leading to unacceptably high rates of healthcare associated infections (HAI), and resulting in unnecessary excess mortality and morbidity in the population and increased healthcare costs due to increased length of hospital stay and more complex care [4].

Guidelines stipulating when HH is required have been in place for many years, but are often not adhered to. Two thirds of the studies included in a systematic review reported compliance rates below 50% [5]. To address the problem of low compliance, many interventions have been designed and evaluated, but the effects are often short lasting or moderate [6]. Grol and Grimshaw made an inventory of the most common interventions used to improve HH practices and described that educational interventions have only short term effects, reminders have a sustained but only modest effect, and performance feedback may be effective, but only if feedback is continued [7]. They concluded that a comprehensive plan, targeting different problems and barriers to change with strategies at different levels (professional, team, patient, and organisation) is needed to achieve lasting changes in HH routines.

It is increasingly recognized that the observed failure to achieve large and sustained effects is due to the absence of well-designed implementation strategies using insights from the behavioural sciences [8,9]. Such strategies are based on a good understanding of factors that contribute to compliance at both the individual and environmental level. Therefore we previously conducted a study that investigated the behavioural correlates of hand hygiene behaviour. This study revealed that the hand hygiene behaviour of physicians and nurses is influenced by different factors (unpublished data Erasmus et al). Contrary to previous interventions, this knowledge should be used in the design of new strategies to improve compliance. But in accordance with the conclusions of Grol and Grimshaw, the study also revealed that factors at other levels than the individual healthcare worker (HCW), including specific barriers for change, should be targeted as well. Even though HCW know they should perform HH in order to protect both themselves and their patients, negative role models, poor accessibility of materials and a poor social culture can hamper good HH [10]. This so-called intention-behaviour gap has been targeted successfully in other fields by using implementation intentions and recently this method has also been applied to hand hygiene improvement [11,12].

The knowledge gained from our previous behavioural research has therefore been used to develop ACCOMPLISH (Actively Creating COMPLIance Saving Health): a multicomponent implementation strategy to improve the hand hygiene behaviour of physicians and nurses. ACCOMPLISH has the potential to substantially increase compliance and reduce HAI in Dutch hospitals, but experimental implementation and evaluation of this package is necessary before large-scale dissemination in healthcare practice can be advised.

## Objectives

This study aims to evaluate the cost-effectiveness of the *ACCOMPLISH *package and will test the effects of this implementation strategy on HH compliance and prevalence of HAI. Furthermore, an economic evaluation will investigate the cost-effectiveness of this strategy from a healthcare perspective.

Research questions:

1. What is the effect of the multicomponent implementation strategy *ACCOMPLISH *on HH compliance rates?

2. Which process indicators contribute most substantially to observed changes in HH compliance?

3. What is the effect of the multicomponent implementation strategy *ACCOMPLISH *on the prevalence of HAI?

4. What is the balance between costs and health effects (costs per prevented HAI) of the *ACCOMPLISH *package?

## Methods/Design

### Study design

**Figure 1:**
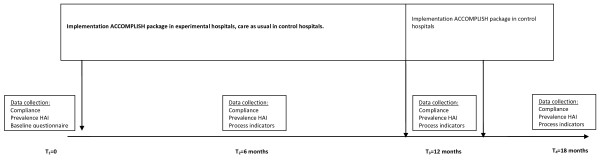
**Study design ACCOMPLISH trial**.

The ACCOMPLISH package will be tested in a two-arm cluster randomized trial in 16 hospitals in the Netherlands (eight intervention and eight control) in one intensive care unit (ICU) and one surgical ward per hospital. In eight hospitals *ACCOMPLISH *will be introduced, while eight control hospitals will maintain normal HH practices. All infection prevention activities as well as any large outbreaks or other major events which might influence HH behaviour will be documented in a process evaluation. The primary outcome measure will be the observed hand hygiene compliance rate, measured at baseline (T1) and after 6 (T2), 12 (T3) and 18 (T4) months. As a secondary outcome measure the prevalence of HAI will be measured at the same time points. At each time point data identifying process indicators will be collected. The process evaluation will also include determinants of implementation, namely the social-political context, the organization, the adopting person (i.e. the HCW), the innovation itself and the facilities needed for implementation [13]. See figure 1 for an overview of the data collection activities.

### Area of study

A multicenter study of 16 hospitals in the Netherlands.

### Sample for study

The sample of 16 hospitals will include university teaching hospitals, large general hospitals, small general hospitals and non-academic teaching hospitals and will de randomised at hospital level. Target population: nurses and physicians with direct patient contact working in the ICU or surgical ward at the participating hospitals in the Netherlands.

## The Intervention

### Intervention program: theoretical background

ACCOMPLISH is evidence and theory based, by following a planned stepwise behavioural research approach to intervention development [14]. It focuses on individual level factors, increasing personal and normative feedback, and is embedded in self-regulation theory, which suggests that behaviour-change is a dynamic process, in which specific, achievable goals need to be set, discrepancies between desired goals and goal progress need to be fed back to an individual, and finally (social) reinforcement is an important element to promote behavioural sustainability and ongoing goal pursuit [11,15]. Among others, it addresses these elements by means of increasing planning, performance feedback, increasing knowledge of HH guidelines on an individual level and on environmental factors creating an increased availability of alcohol based hand rub and increasing the visibility of HH guideline support. These factors were identified as major determinants of non-compliance in a previous study (unpublished data, Erasmus et al.).

The ACCOMPLISH package includes changes to the physical and social environment, performance feedback & goal setting, training and action planning (see table 1):

**Table 1:** Content of the intervention, including operationalisation and targeted behavioural correlate

Content of intervention	Operationalised as	Target behavioural correlate
Physical environment	Electronic alcohol based hand rub dispenser	Habit, availability of materials

	Posters and reminders in workplace	Raise awareness

Social environment	Interactive team training sessions	Social and descriptive norms, goal setting, role models

Education	E-learning modules	Factual knowledge, risk perception, action plans, planning

Performance feedback	Feedback of frequency data: monthly reports with reward	Social norms, self-regulation

1. The physical environment will be adapted by improving the availability of alcohol based hand rub by electronic dispensers with an electronic feedback system. These dispensers will enable easy access and also remind HCW to perform HH at the point of care. The dispensers register each use with a time stamp, which will be used for periodic feedback.

2. The social environment will be targeted with training at group and individual level based on the Helping Hands team training package, to improve social and descriptive norms. [Huis *et al. Helping Hands: a cluster randomised trial to evaluate the effectiveness of two different strategies for promoting hand hygiene in hospital nurses, submitted*] The unique contribution of this part of the intervention is built upon the Social Learning Theory,[16] the Social Influence Theory,[17] the Theory on Team Effectiveness,[18] and the Leadership Theory [19]. The key elements of the training are: gaining active commitment and initiative of ward management and team members, modelling by informal leaders at the ward, setting norms and targets within the team, identifying barriers and formulating activities to improve. This team training will be delivered to nurses and physicians separately. The physician teams will receive one interactive group session, and a web-based session for individual completion a month later. The nurse teams will receive three interactive team sessions during a period of 3-4 months. The interactive sessions will be led by a specially trained coach. During the intervention period the ward manager actively supports the team.

3. Performance feedback is an effective tool to increase awareness, and has often been effective in improving HH for short periods [20]. Through periodical performance feedback of HH frequencies, as measured by the novel dispenser mentioned above, a sustained positive effect on hand hygiene behaviour could be accomplished. Different mediums will be used for communicating the feedback (i.e. newsletters, electronic feedback) and will be imbedded into pre-existing work routines. A reward programme will be linked to the performance feedback in order to provide positive reinforcement of the desired behaviour (i.e. ward with highest increase in compliance).

4. An e-learning module will be used for education purposes, focussing on increasing factual knowledge, risk perception and the formulation of action plans. The module will be used for both physicians and nurses, but tailored to their specific behavioural correlates. Planning will be increased by making action plans (implementation intentions), as part of the training in which HCW will be assisted in making concrete plans and formulating solutions to the everyday problems they encounter concerning HH, using if-then plans. Concretely, it will involve formulating plans how and when they intend to perform hand hygiene (i.e. if I am about to examine a patient in the ICU, then I first walk past the alcohol based hand rub dispenser), as has been successfully used in other areas of public health [21] and more specifically for improving hand hygiene [12].

### Implementation

In order to increase the chances of successful implementation, an extensive communication strategy will be put in place prior to the start of the intervention period.

In each hospital contact persons will be appointed: the Infection Control Practioner (ICP), who will act as a coordinator for all activities, and also as the primary contact between the researchers and hospital. Other contacts will include medical staff, nurses and managers form each hospital, to stimulate active participation in the study by all groups.

At the start of the intervention a festive kick-off meeting will be planned in the participating units, in which HCW will be informed about the intervention activities. Special attention will be paid to the reasons for a process evaluation during the study. HCW will be instructed as to how they can signal when a component of the intervention is interfering with their work activities, so that effective measures to solve these problems can be set in motion swiftly. Twelve months after the start of the intervention post intervention measurements will commence.

After the post-intervention measurements, the ACCOMPLISH package will be adjusted for all process evaluation information gathered and will be offered to the control hospitals. Based on experience gained from the study, they will receive instruction on how to implement the package.

## Outcome Measures

### Primary outcome measure: observed compliance rate

The compliance rate is operationalised as the number of HH practices divided by the number of opportunities for HH according to the international guidelines of the WHO [22], which state that HH must be performed before and after patient contact, after contact with patient surroundings, after body fluid exposure risk, and before aseptic tasks. Compliance will be assessed covertly by direct observation at T1(baseline), T2 (6 months post-intervention), T3 (12 months post intervention) and T4 (18 months post intervention) at the individual level by a trained observer with the electronic Dutch version of the Hand hygiene Observation Instrument [23]. All observations will be collected between 7:30 and 13:00. Each observation session will last 4-5 hours, during which time at least three different nurses will be followed in their duties. Furthermore, any physicians caring for the same patient as the nurse being followed will also be observed.

Although some HCW may be aware of the true nature of the study, most will revert to their routine quickly, reducing the Hawthorne effect [24]. In addition, the frequency of HH per ward will be collected continuously by the novel electronic dispensers. Furthermore, data on environmental factors influencing HH will be collected (such as patient to nurse ratio and the number of patients on the ward).

### Secondary outcome measure: prevalence of HAI

The prevalence rate is operationalized as the number of HAI per 100 patients. This will be assessed with the PREZIES module of the national surveillance system of HAI during the 18 month intervention period (http://www.prezies.nl/). This module is comparable to CDC surveillance methods (http://www.cdc.gov/HAI/surveillance/monitorHAI.html). Point-prevalence will be measured at T1, T2, T3 and T4 in all experimental and control units, in a given time slot (two week period). All prevalence data will be collected by a qualified ICP, who will be trained before commencement of the study. At each data collection point two point-prevalence measurements will collected, and the data of the two measurements averaged to adjust for fluctuations.

## Process indicators

In order to identify barriers and facilitators of the implementation of ACCOMPLISH, information on process indicators will be collected at T1, T2, T3 and T4. Special attention will be paid to the different types of determinants of innovation in healthcare, which can be divided into 5 groups, namely the socio-political context, the organization, the adopting person or HCW, the innovation and the facilities needed to implement the innovation [13]. Information will be collected on HCW experiences with components of the intervention, on self-reported compliance rates and behavioural and environmental determinants of non-compliance using a questionnaire based on the Theory of Planned Behaviour in addition to constructs identified in qualitative research [10,25]. It will provide information about dissemination requirements, needed for large scale application. Other process indicators identifying possible infection prevention activities outside the intervention package will be collected during the entire intervention.

## Economic Evaluation

An ex-post economic evaluation of the ACCOMPLISH package to improve compliance to hand hygiene guidelines compared to a control group, from a healthcare perspective will be performed in accordance with the Dutch guidelines [26].

A cost-effectiveness analysis will assess the balance between costs of the intervention and effects of improved HH, from a healthcare perspective. Costs for all separate actions and time used by all individual healthcare professionals, the training program, costs for the electronic alcohol based hand rub dispensers and all other materials will be measured from a healthcare perspective for the intervention package. Table 2 gives an overview of the costs which will be calculated.

**Table 2:** Costs to be calculated for cost-effectiveness analysis

Intervention component	Costs to be calculated
Alcohol dispensers	1. costs of alcohol rub dispenser (purchase + maintenance)
	2. costs of placement (staffing costs)
	3. costs of more frequent refilling due to increased use of alcohol hand rub (compared to control unit)
	4. costs of alcohol hand rub due to increased use (compared to control unit)
	5. extra staffing time needed to perform HH compared to control unit (# times HH is performed extra X average time needed for HH as measured by new dispenser)

Feedback component	1. promotional materials for feedback element (posters, newsletters)
	2. costs for rewards
	3. extra time needed to incorporate feedback into daily work activities (i.e. during existing staff meetings)

Training component	1. production costs social and web based training
	2. maintenance costs web based training
	3. staffing costs for staff time needed to participate in training sessions

Overall	1. staffing costs continuous maintenance intervention components by infection control nurse
	2. training infection control nurse prior to intervention

For the calculation of the saving due to reduced healthcare use of patients without HAI total intramural medical costs of comparable patients with HAI will be calculated.

For the most important cost items, unit prices will be determined by following the micro-costing method [27], which is based on a detailed inventory and measurement of all resources used. Resource costs arise within the hospital and consist of outpatient visits, inpatient days, use of the operation room, radiology examinations, blood tests, etc. Real medical costs will be calculated by multiplying the volumes of healthcare use with the corresponding unit prices. For instance, the calculation of the costs of complications and prolonged hospital stay will consist of detailed measurement of investments in manpower, equipment, materials, housing and overhead. The salary schemes of hospitals and other healthcare suppliers will be used to estimate costs per hour for each healthcare professional. Taxes, social securities and vacations will be included.

Data on effects (reduction of HAI), costs (time costs of extra HH and material and development costs) and savings (reduced healthcare use of patients without HAI vs. comparable patients with HAI) will all be collected in this study. Data on treatment (hospitalisation) and follow-up consultations will be collected retrospectively from (electronic) patient charts and hospital administrations in a case-control study (60 cases, 60 controls). From each of the 4 strata (university teaching, non-academic teaching, large general, small general) 1 hospital will be selected for the case control study, and from this hospital 15 cases will be selected by the local ICP. Fifteen controls will be matched by characteristics such as age, sex and diagnosis on admittance. Since cost data per patient (but not per day care) are typically highly skewed, nonparametric bootstrap techniques will be used for calculating the differences in distributions of the direct medical costs. The data will be extracted from the patient files by a researcher using an anonymous data-collection form. Information will be collected on, among others, length of hospital stay, length of ICU stay, number of days on artificial ventilation, number of intravasal or urinary catheters placed and on antibiotic use.

To measure the economic impact of improved hand hygiene cost-effectiveness will be assessed by calculating the incremental cost-effectiveness ratio, defined here as the costs for the intervention (minus savings) divided by the difference in prevalence of HAI between the intervention and control group. Costs and effects will both be discounted with a 3% ratio. A sensitivity analysis will be performed to demonstrate the sensitivity of the results to changes in improved compliance under influence of process indicators (barriers and facilitators). Prognostic modelling will be used for a limited set of predictors of outcome (e.g. attendance of training sessions, alcohol hand rub availability, and frequency of feedback sessions by ICP).

### Sample size calculations

Compliance: Compliance rates are expected to increase from 20% at baseline to 50% twelve months after the intervention. Assuming 120 observed opportunities for compliance per ward per measurement moment and a 10% heterogeneity between wards, a power of 93% was calculated with two-sided alpha = 0.05 for this design. In each hospital one ICU and one surgical ward will be included in the study.

Prevalence of HAI: 8568 patients are expected to be included in this study. Based on PREZIES data a baseline prevalence of HAI of 12% in the surgical ward and of 25% in the ICU is estimated [28]. Approximately 30% of these infections are exogenous and based on international literature a reduction of 40% of the exogenous part of these HAI can be expected in the intervention hospitals [29]. This will lead to a reduction in HAI in the surgical ward from 12-> 10%, and in the ICU from 25->22%. Based on an average reduction of 16-> 14% (when pooling the data) a power of 73% with a two-sided alpha = 0.05 was calculated. Since baseline measurements will be collected in all units this will enable comparison within units, substantially increasing the power.

### Selection of the sample

Randomisation will take place at hospital level by means of computer generation. Therefore in each hospital both wards will be included in the same arm of the trial.

### Data analysis

Process indicator questionnaires: These will be analysed with multivariable linear regression analysis, with self-reported compliance (never-always on a 10-point scale) as primary outcome variable. The determinants included in the questionnaire will include the socio-political context, the organization, the adopting person or HCW, the innovation and the facilities needed to implement the innovation [13]. Furthermore behavioural determinants will be included in the questionnaire, including attitudes, social norms, perceived behavioural control, intention, habit, knowledge of the guidelines and patient safety culture (as measured by the ComPaz questionnaire) [30].

Randomised controlled trial: The effect of the intervention strategy on the overall compliance rate among HCW (logistic regression analysis), and on prevalence of HAI will be assessed. Multilevel analysis will be performed to compensate for the clustered nature of the data (HCW are clustered within wards), using mixed linear modelling techniques.

## Discussion

In this paper the ACCOMPLISH intervention study has been outlined. This study is the first RCT to investigate the effects of a HH intervention programme on the number of HAI, and the first to investigate the cost-effectiveness of such an intervention. Furthermore, this is one of the first interventions tailored to the different behavioural determinants of physicians and nurses.

Strengths of the study are number of participating centres, the cluster randomized controlled trial design and the length of follow-up (6, 12 and 18 months). Furthermore, the inclusion of not only a process measure (compliance) as outcome, but also the number of HAI and the cost-effectiveness study add to the strength of the study design. The limitations of the study are the use of prevalence rather than incidence data and the fact that that not all process evaluation instruments have been validated.

The results of the study will contribute to the body of evidence on influencing HH and shed light on the cost-effectiveness of such measures from a societal perspective. In addition, if the ACCOMPLISH package proves success in improving HH compliance and lowering the prevalence of HAI the package could be disseminated at national and international level.

## Ethical considerations

The study protocol has been approved by the Medical Ethics Board of the Erasmus University Medical Centre in Rotterdam. In the Netherlands is it not necessary to collect informed consent from healthcare workers before observing their behaviour, although all participating wards were informed about the study prior to commencement.

## Competing interests

The authors declare that they have no competing interests.

## Authors' contributions

VE, EB, and MV conceived of the study and its design and drafted the manuscript. ES, PE and SP participated in the design of the study and revised the manuscript. AH, AO, MB, EB and JR revised the manuscript for important intellectual content. All authors read and approved this final manuscript.

## Pre-publication history

The pre-publication history for this paper can be accessed here:

http://www.biomedcentral.com/1471-2458/11/721/prepub
